# Ghrelin is a prognostic marker and a potential therapeutic target in breast cancer

**DOI:** 10.1371/journal.pone.0176059

**Published:** 2017-04-18

**Authors:** Malin Grönberg, Cecilia Ahlin, Ylva Naeser, Eva Tiensuu Janson, Lars Holmberg, Marie-Louise Fjällskog

**Affiliations:** 1 Department of Medical Sciences, Section of Endocrine Oncology, Uppsala University, Uppsala, Sweden; 2 Department of Oncology, Faculty of Medicine and Health, Örebro University, Örebro, Sweden; 3 Department of Surgical Sciences, Uppsala University, Uppsala, Sweden; 4 Faculty of Life Sciences and Medicine, King’s College London, London, United Kingdom; University of South Alabama Mitchell Cancer Institute, UNITED STATES

## Abstract

Ghrelin and obestatin are gastrointestinal peptides, encoded by the same preproghrelin gene. Both are expressed in breast cancer tissue and ghrelin has been implicated in breast cancer tumorigenesis. Despite recent advances in breast cancer management the need for new prognostic markers and potential therapeutic targets in breast cancer remains high. We studied the prognostic impact of ghrelin and obestatin in women with node negative breast cancer.

Within a cohort of women with breast cancer with tumor size ≤ 50 mm, no lymph node metastases and no initiation of adjuvant chemotherapy, 190 women were identified who died from breast cancer and randomly selected 190 women alive at the corresponding time as controls. Tumor tissues were immunostained with antibodies versus the peptides.

Ghrelin expression was associated with better breast cancer specific survival in univariate analyses (OR 0.55, 95% CI 0.36–0.84) and in multivariate models, adjusted for endocrine treatment and age (OR 0.57, 95% CI 0.36–0.89). Obestatin expression was non-informative (OR 1.2, 95% CI 0.60–2.46). Ghrelin expression is independent prognostic factor for breast cancer death in node negative patients—halving the risk for dying of breast cancer. Our data implies that ghrelin could be a potential therapeutic target in breast cancer treatment.

## Introduction

Ghrelin is a 28 amino acid peptide which was first identified as a ligand of growth hormone (GH) secretagogue receptors (GHSRs) that regulates GH secretion [1]. It is a multifunctional peptide with physiological actions ranging from hormonal secretion, regulation of food intake, modulation of insulin secretion, adipogenesis and gastrointestinal motility, and one of the most important orexigenic peptides currently known [1–5]. It’s involvement in cell proliferation [6–8] and milk production stimulation [9], together with the stimulatory effect on GH levels by the ghrelin axis [1], and its high levels in females [10], gives ghrelin a possible role in tumorigenesis [11]. Moreover, obesity, a well-known breast cancer risk factor, is strongly correlated with low ghrelin levels [12, 13]. Obestatin, a 23 amino acid peptide hormone which was discovered in 2005, is another ghrelin-gene derived peptide and was initially suggested to be a functional ghrelin antagonist, a proposal that has been disputed [14, 15], and its physiological functions are still controversial.

Many of the ghrelin system constituents (ghrelin, obestatin, ghrelin splicing variants, and GHSRs) are present in normal breast tissue, breast tumors, and breast cancer cell lines [16–18]. In a prior study, expression of ghrelin was associated with a positive outcome in a non-consecutive and selected patient population of invasive breast tumors, demonstrating a 3-fold lower risk for breast cancer death in patients with tumors expressing ghrelin compared to those lacking ghrelin expression [19]. Moreover, is has been reported that an increase in breast cancer risk is correlated with various ghrelin gene polymorphisms [20]. A difference in regulation of the ghrelin system in breast tissue might therefore influence pathological breast development. In line with that, ghrelin gene-derived splice forms are also overexpressed in breast cancer [18, 21, 22].

Furthermore, specific receptors which bind natural ghrelin and synthetic GH secretagogues (GHSs) are present in human breast carcinomas. This binding is independent of tumor histological type, stage, ER status, Ki67, pre- or postmenopausal status, but correlates to the grade of tumor differentiation [6]. Well differentiated carcinomas show a higher GHS binding compared to moderately and poorly differentiated carcinomas [6].

Studies on the functional role of the ghrelin system in the regulation of relevant processes in breast cancer development and progression are still limited and data are conflicting. Ghrelin causes significant inhibition of cell proliferation in human breast carcinoma cell lines and thus, may have a clinical application in breast cancer therapy [6–8]. Consistent with an inhibitory role of ghrelin in breast cancers, a previous study show that ghrelin expression is correlated to low histologic grade, estrogen receptor positivity, small tumor size, and low proliferation of human breast tumors [19]. However, also proliferation promoting effects have been observed [18].

The ghrelin system may be attractive target for new treatment possibilities. Potential therapeutic approaches using ghrelin mimetic compounds and antagonists in clinical disease are currently being developed. The orexigenic and GH releasing effects of ghrelin make it a good agent to be used in catabolic states/situations. In recent years, synthetic agonists and antagonists of the ghrelin receptor have been developed for the possible treatment of metabolic or nutritional disorders [23, 24]. The ghrelin/GH axis seems to be involved in the breast cancer tumorigenesis, although a precise role has not been yet established [22]. As both anti-proliferatve and proliferative effects has been found, there is still some hesitation around the usefulness of ghrelin agonists as potential therapeutic options in breast cancer.

In the present study, we examined the roles of ghrelin and obestatin as prognostic factors in a clinically well-characterized set of cases and controls nested within a population based cohort of lymph node negative, chemotherapy-naive patients.

## Material and methods

### Subjects

The source population of the study was a defined cohort of women diagnosed with breast cancer in the Uppsala-Örebro region, Sweden, from 1993–2004. Information about the patients was derived from the Uppsala-Örebro Breast Cancer Register, which is a population-based clinical database with coverage of over 98%. Inclusion criteria for this study were tumor size ≤50 mm, no lymph node metastases, and no adjuvant chemotherapy. Nine hundred women met the inclusion criteria. Within this cohort, cases were defined as women who died from breast cancer as reported to the national causes of death register. All eligible cases were selected. Eligible as controls were women in the cohort alive at the time of the corresponding case's death. Two-hundred and forty cases were identified. For each identified case, one control was randomly selected. Fifty cases and corresponding controls were excluded from the study for not meeting inclusion criteria after reviewing data from patient records and pathology reports, or because of missing tumor blocks: 26 patients had new/contralateral or locally advanced breast cancer, in 12 patients no paraffin blocks were found, six patients had non-breast cancer death, four patients had distant metastases at diagnosis, one patient received adjuvant chemotherapy, and one patient had no breast surgery performed. This resulted in a total of 190 cases and 190 controls. The average age was 66 years for cases and 61 years for controls. The average tumor size was 20 mm for cases and 16 mm for controls. All patients underwent surgery consisting of either modified radical mastectomy with axillary dissection, or sector resection with axillary dissection and post-operative irradiation of the breast. Fifty-three (28%) cases and 48 (25%) controls received antihormonal therapy. Patients’ characteristics including grade, hormone receptors and HER2 are shown in Table 1.

**Table 1:** Patients’ characteristics.

Parameter		Case, n (%)	Control, n (%)
Age, average (years)		66	61
Tumor size, average (mm)		20	16
Tumor histology			
	Ductal	163 (86)	145 (76)
	Lobular	20 (10)	23 (12)
	Others	7 (4)	22 (12)
Histologic grade			
	I	19 (10)	48 (25)
	II	94 (50)	105 (55)
	III	76 (40)	34 (18)
	Not known	1 (0)	3 (2)
ER			
	Positive	103 (54)	147 (77)
	Negative	79 (42)	41 (22)
	Not known	8 (4)	2 (1)
PgR			
	Positive	73 (38)	127 (66)
	Negative	108 (57)	60 (32)
	Not known	9 (5)	3 (1)
Ki67			
	High (>22%)	80 (42)	56 (29)
	Low	100 (53)	127 (67)
	Not known	10 (5)	7 (4)
HER2			
	Overexpression (IHC 3+ or FISH pos)	18 (10)	13 (7)
	Normal	158 (83)	161 (85)
	Not known	14 (7)	16 (8)
Adjuvant radiotherapy			
	Yes	101 (53)	116 (61)
	No	89 (47)	74 (39)
Adjuvant endocrine therapy			
	Yes	53 (28)	47 (25)
	No	137 (72)	143 (75)
Total		190	190

ER, estrogen receptor; FISH, fluorescent *in situ* hybridization; HER2, human epidermal growth factor receptor 2; IHC, immunohistochemistry; PgR, progesterone receptor.

The study was approved and the need for consent was waived by the local ethics committee, Regionala etikprövningsnämnden (EPN), in Uppsala, Sweden.

### TMA construction

Tissue microarrays (TMA) were produced at the TMA facility, the Department of Laboratory Medicine, Center for Molecular Pathology, Lund University. Paraffin blocks from the patients' primary tumor were collected. Hematoxylin and eosin sections were reviewed and areas with invasive tumor were selected. Each tumor was reevaluated and reclassified according to the Elston and Ellis grading system (R-MA) [25]. Representative areas from each tumor were punched and brought into recipient paraffin blocks to construct TMAs consisting of two cores (diameter 1 mm) of each tumor. Then, 3–4 μm thick sections were cut from array blocks and transferred to glass slides.

### Antibodies

The primary antibodies used for immunohistochemical staining were anti-obestatin, an in-house developed antibody (rabbit polyclonal), for which the production and characterization has been described previously [26] and anti-ghrelin (Rabbit polyclonal, H-031-30, lot no 01298–1, Phoenix Pharmaceuticals, Belmont, CA, USA). Both antibodies were diluted 1:2000.

### Immunohistochemistry

Immunohistochemical staining was performed using the Dako EnVision Plus-HRP Detection Kit (K401111-2, Dako, Glostrup, Denmark) according to the manufacturer’s instructions. For antigen retrieval the sections were subjected to pre-treatment (microwave heating for 10 min at 750 W followed by 15 min at 380 W using Tris-HCl buffered saline, pH 8.0). The sections were incubated with the primary antibodies in PBS with 1% BSA over night at 4°C. Bound antibodies were visualized by incubation with liquid 3, 3′-Diaminobenzidine substrate chromogen for 5 min.

The analyses of the ghrelin and obestatin immunostainings were manually performed by two observers (MG and YN). None of the investigators had access to clinical data or outcomes. The intensity of the staining in the tumor cells was examined and scored on a scale as non-immunoreactive (non-IR, 0), weak (1), moderate (2) and strong (3). Each of the two cores from every tumor on the array was examined and scored separately. In case of conflicting results, a third evaluation was performed by the observers and consensus was reached. If one of the two tumor cores was lost, the remaining one was used for scoring. The entire core(s) from each tumor was examined and at least 200 tumor cells had to be evaluable to be designated an intensity score. Positive staining was defined as complete and/or partial (>50% IR tumor cells) staining at any intensity that could be differentiated from truly negative staining, background and diffuse non-specific staining. Cytoplasmic staining in high-power fields (40X objective) was accepted as positive reaction.

Photographs were taken using a Zeiss Observer Z1 microscope and the Axiovision software (Carl Zeiss, Gottingen, Germany).

This material has been used previously [27] and staining procedures and scoring of ER, PgR, Ki67, HER2 and Nottingham histological grade (NHG) of the material have been previously described [28].

### Immunohistochemical controls

The specificity of the ghrelin and obestatin antibodies has been evaluated and presented previously [26]. Normal human gastric mucosa was used as positive control.

### Statistical analyses

Conditional logistic regression analysis was performed to estimate odds ratios (ORs) and confidence intervals (CIs) as a measure of the relative risk of being at decreased or increased risk of dying from breast cancer due to having a breast cancer with ghrelin and/or obestatin expression. Established prognostic factors such as age, tumor size and histological grade as well as ghrelin and obestatin were analyzed in univariate models.

Adjustment for adjuvant endocrine therapy and age was also performed. Correlations between ghrelin/obestatin and other clinicopathological parameters were assessed with Spearman's correlation test.

A directed acyclic graph (DAG) was used to determine factors to include a multivariate model, indicating that none of the factors should be included in the model [29]. DAGs are frequently used to represent independence and relationships among variables in a complex system [30, 31].

Comparison of the agreement of the two observers’ results was performed to evaluate the reproducibility of the scoring of the immunohistochemical results. The material was manually scored using a light microscope. The degree of concordance between the two investigators was quantified as the chance-corrected measure of agreement, known as kappa [32]. The commonly applied definition for the interpretation of different kappa values that was used here is as follows: 0.01–0.20 as none to slight; 0.21–0.40 fair; 0.41–0.60 moderate; 0.61–0.80 substantial; 0.81–1.00 as almost perfect agreement. All statistical analyses were performed using IBM SPSS Statistics software (v21, USA).

## Results

### Immunoreactivity in breast tumor samples

Results from routine stainings for hormone receptors and HER2 are described in the patients’ characteristics (Table 1). Of 380 case-controls analyzed on the TMAs, data were missing from 25 tumors (6.6%) for ghrelin and 30 tumors (7.9%) for obestatin. Loss of some data occurred due to having too little evaluable tumor tissue. The results from the immunohistochemical stainings are presented in Table 2.

**Table 2:** Results from the immunohistochemical stainings.

	Case, n (%)	Control, n (%)	Total, n (%)
**Ghrelin intensity**			
0	113 (64)	88 (49)	201 (57)
1	38 (21)	41 (23)	79 (22)
2	18 (10)	34 (19)	52 (15)
3	8 (5)	15 (9)	23 (6)
Total, n	177	178	355
**Obestatin intensity**			
0	17 (9)	19 (11)	36 (10)
1	21 (12)	14 (8)	35 (10)
2	66 (37)	55 (32)	121 (35)
3	74 (42)	84 (49)	158 (45)
Total, n	178	172	350

The intensity of the staining in the tumor cells scored on a scale as non-immunoreactive (0), weak (1), moderate (2) and strong (3).

**Fig 1:**
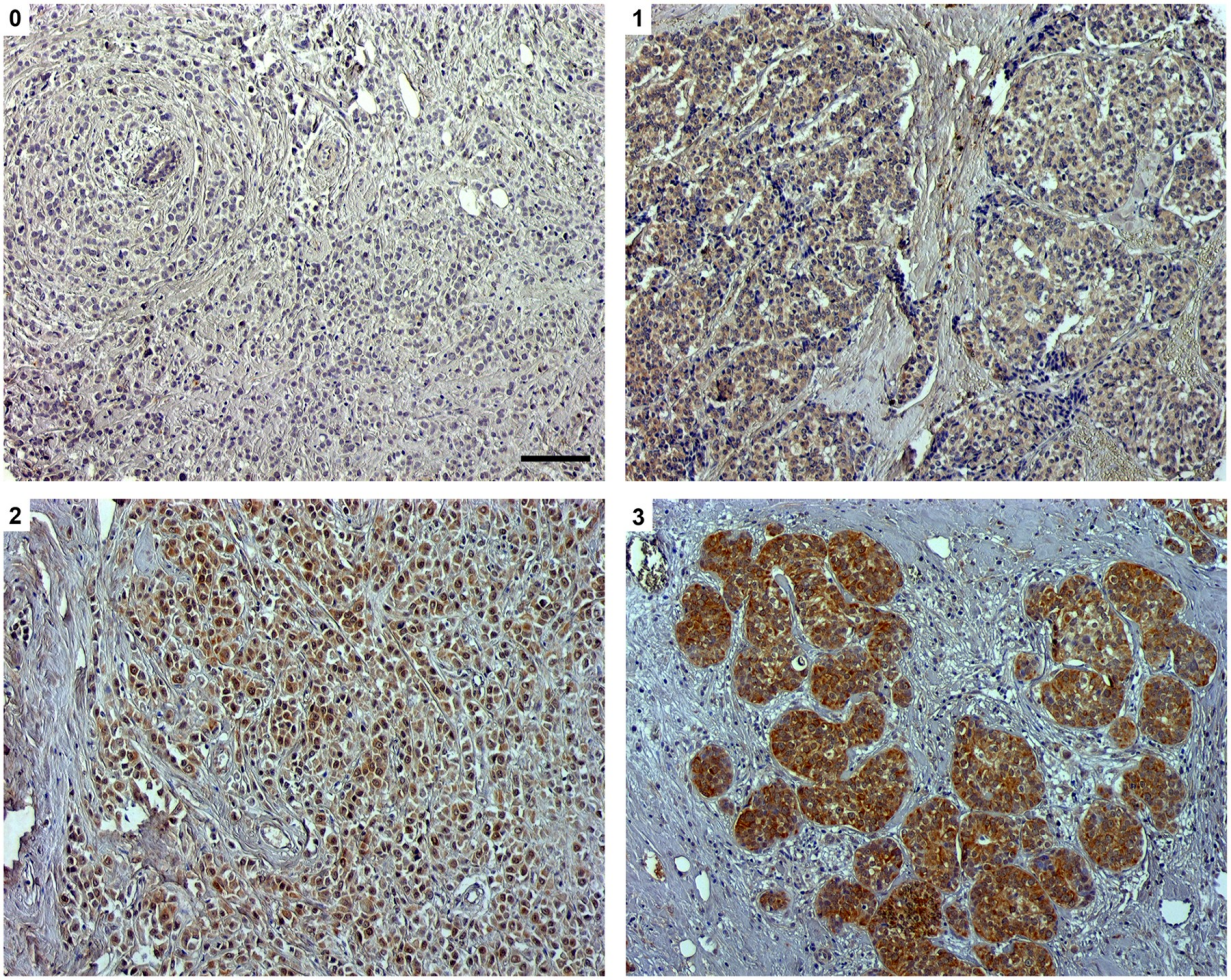
Ghrelin expression in node negative breast cancer tissue was analyzed by immunohistochemistry. Representative images of ghrelin with 0 (non-immunoreactive), 1 (weak), 2 (moderate) and 3 (strong) immunostaining. Scale bar = 100 μm.

Various patterns of immunostaining intensity were observed. The majority of the cases/controls had a low to moderate intensity in the ghrelin stainings. A tendency towards a higher intensity in the obestatin stainings was observed. Representative photos from the ghrelin immunostainings are shown in Fig 1.

### Correlations between ghrelin/obestatin to each other and other clinicopathologic parameters

Ghrelin and obestatin were positively correlated to each other (Spearman correlation). No strong correlations could be identified between ghrelin/obestatin to any of the investigated clinicopathological parameters. Obestatin had statistically significant correlations with ER, NHG and Ki67, but still at negligible levels with Spearman correlation coefficients <0.20. Spearman’s correlations are presented in Table 3.

**Table 3:** Ghrelin and obestatin expression in node-negative breast cancer in relation to clinicopathologic variables.

Ghrelin	ρ	p-value	n
Tumor size	-0.01	0.87	178
Age	-0.14	0.06	178
ER	0.10	0.20	176
PgR	0.12	0.10	176
HER2	-0.05	0.50	164
NHG	-0.12	0.12	176
Ki67	-0.12	0.11	174
**Obestatin**			
Tumor size	-0.09	0.26	172
Age	0.02	0.83	172
ER	0.18	0.02	170
PgR	0.11	0.16	170
HER2	0.04	0.58	158
NHG	-0.16	0.03	169
Ki67	-0.17	0.03	167
Ghrelin	0.22	0.004	165

ER, estrogen receptor; HER2, human epidermal growth factor receptor 2; NHG, Nottingham Histological Grade; PgR, progesterone receptor; ρ, Spearman’s correlation test coefficient.

### Risk of breast cancer death

A statistically significant association was observed between breast cancer death and expression of ghrelin (p-value 0.006), histological grade (p-value 0.00), age (p-value 0.00) and tumor size (p-value 0.001) using conditional logistic regression in a univariate model. Overexpression of HER2 was not statistically associated with breast cancer death (p-value 0.83), neither was obestatin (p-value 0.59). The numerically highest ORs for breast cancer death were predicted by grade (OR ≥ 3). OR for breast cancer death using Ki67 was numerically lower (OR < 2). Data is summarized in Table 4.

**Table 4:** Univariate and multivariate analysis of prognostic parameters of breast cancer death.

		OR (95% CI)	p-value
**Univariate analysis**			
Tumor size	≤20 mm vs. >20 mm	2.2 (1.3–3.5)	0.001
NHG	I+II vs. III	3.0 (1.8–4.9)	0.00
Ki67	≤22% vs. >22% [28, 33]	1.7 (1.1–2.6)	0.02
HER2	Negative vs. positive	0.9 (0.4–2.1)	0.83
Age	<70 yrs vs. ≥70 yrs	2.4 (1.5–3.9)	0.00
Ghrelin intensity	0 vs. 1+2+3 [19]	0.6 (0.4–0.8)	0.006
Obestatin intensity	0 vs. 1+2+3 [19]	1.2 (0.6–2.5)	0.59
**Multivariate analysis**			
Ghrelin intensity*	0 vs. 1+2+3 [19]	0.6 (0.4–0.9)	0.007
Endocrine treatment	Negative vs. positive	1.0 (0.6–1.7)	0.93
Ghrelin intensity**	0 vs. 1+2+3 [19]	0.6 (0.4–0.9)	0.01
Endocrine treatment	Negative vs. positive	1.0 (0.6–1.7)	0.95
Age	<70 yrs vs. ≥70 yrs	2.4 (1.4–3.9)	0.001

Odds ratio (OR) and 95% confidence intervals (CI) obtained from univariate and multivariable Cox conditional logistic models. HER2, human epidermal growth factor receptor 2; NHG, Nottingham Histological Grade.

*Model adjusted for endocrine treatment.

**Model adjusted for endocrine treatment and age.

The DAG analysis showed that none of the factors influenced ghrelin in such way that they should be included in a multivariate model. To further exclude confounding, two additional models were performed. An analysis on ghrelin adjusted for endocrine treatment showed no change in the prognostic power of ghrelin with an OR of 0.55 (95% CI, 0.36–0.85) in the adjusted analysis as compared with OR 0.55 (95% CI, 0.36–0.84) in the non-adjusted analysis. Thus, endocrine treatment did not appreciably affect the prognostic value of ghrelin. In a model adjusted for both endocrine treatment and age, ghrelin was still a prognostic factor with OR 0.57 (95% CI, 0.36–0.89).

### Reproducibility

The two investigators (MG and YN) examined the ghrelin and obestatin immunostainings “blindly” and separately. The kappa value for ghrelin was 0.81 and for obestatin 0.83 (almost perfect agreement), demonstrating very good reproducibility of the scorings.

### Control experiments

In all experiments the positive control showed IR cells in the deeper third part of the gastric mucosa as expected (data not shown).

## Discussion

This study shows that expression of ghrelin is a prognostic factor for lymph node negative breast cancer patients, not treated with adjuvant chemotherapy, in both uni-and multivariate analyses.

The prognostic value of ghrelin and obestatin was assessed in a well-defined group of women with breast cancer as the study was designed with the intention to investigate potential prognostic factors in breast cancer. The number of cases (breast cancer deaths), i.e., the events that drive the statistical power, is high for a study in the field. Thus, our data should reflect results that would have been achieved if the whole cohort had been studied.

One limitation of this study is that women received adjuvant endocrine treatment according to standard guidelines spanning over a time period of more than 20 years. Endocrine treatment was inconsistently prescribed, where 73% of the women receiving adjuvant endocrine treatment had ER positive tumors and the rest (27%) ER negative tumors**,** according to our repeated ER stainings, and only 31% of all patients with ER positive tumors received adjuvant endocrine treatment.

To determine which factors to be included in the multivariate analysis, DAG depicting causal relations was used. This was done to assess whether adjustment for a factor minimizes or introduces bias and to select possible confounding factors where causal relations are depicted. Clinicopathologic factors included in this study, such as ER, proliferation index and age, are all well-known factors associated with breast cancer survival. To our knowledge, there is no indication that ghrelin influences these factors in such a way that it in turn would affect breast cancer prognosis; hence, they should not be included in the statistical model. This is in line with the results from the correlation analyses/DAG, where none of the factors is strongly correlated to ghrelin. Additional analyses to further exclude the risk of confounding showed that ghrelin was a prognostic factor independent from endocrine treatment and age.

The data from this study suggest that patients with tumors non-IR for ghrelin have approximately 2 times higher risk for breast cancer-specific death, similar to established prognostic factors, giving ghrelin a possible role as a new prognostic marker. The prognostic importance of ghrelin seems robust since we have obtained similar results in another study [19]: ghrelin expression correlated to a better breast cancer survival for women with invasive breast cancer (*n* = 144). Ghrelin and ghrelin gene-derived peptides, have been documented in breast cancer studies [16, 18–20, 22]. However, comparable studies on ghrelin in breast cancer are still limited, making comparisons to this study difficult due to different techniques, aims and/or antibodies used. A study by Jeffery *et al*. [18] showed that ghrelin was present in low levels in normal breast tissue, with moderately higher levels of staining in breast cancer samples. However, the possible prognostic value of ghrelin was not studied here. In addition, the preproghrelin isoform exon-3-deleted ghrelin was more highly expressed in breast cancer compared to normal breast tissue, the highest level was detected in grade 3 breast carcinoma. In another study [22], using qRT-PCR, it was shown that expression levels of ghrelin did not differ between normal breast tissue and breast cancer. However, levels of the ghrelin variant In1-ghrelin was 8-times higher in breast cancer tissues than in normal mammary tissue. Taken together, ghrelin and ghrelin-gene derived peptides and variants seem to be differently expressed in normal breast tissue and cancer. The relevance of this still needs to be elucidated. Comparable studies evaluating the prognostic role of ghrelin in breast cancer are still lacking.

In this study, obestatin did not provide any prognostic information as opposed to ghrelin, despite being derived from the same precursor. Although there was a significant correlation between ghrelin and obestatin, it was weak (rho value 0.22). Thus, it was anticipated that the two proteins would not provide different similar prognostic information. Nonetheless, the result is in accordance with our previous studies [19]. One explanation for the different protein distribution of the peptides in the same tumor could be the complexity of the ghrelin gene [34]. A revision of the structure of the human ghrelin gene has demonstrated the presence of novel exons, alternative splice variants together with novel transcripts encoding C-ghrelin and a transcript encoding only for obestatin. This suggests that ghrelin gene-derived peptides may also be produced independently of preproghrelin [34]. Furthermore, the antibodies used in this study, ghrelin (Phoenix Pharmaceuticals) and obestatin (designed by our research group), are directed towards obestatin and ghrelin, which are the cleavage products of preproghrelin. The ghrelin antibody recognizes both acylated and non-acylated ghrelin. The obestatin antibody was developed using a 23 amino acid peptide, identical with human obestatin with an additional amino-terminal cysteine residue. However, we cannot rule out the possibility that in some instances the ghrelin and obestatin antibodies detect the preproghrelin peptide. Further evaluation using other antibodies versus other transcripts may be helpful.

The results from this study indicate that ghrelin could be used as a prognostic factor in breast cancer. Additionally, ghrelin represents an interesting and attractive target [35, 36], playing fundamental roles in maintaining metabolic homeostasis, GH levels and body composition, and could therefore provide new treatment alternatives in breast cancer.

In conclusion, ghrelin is a prognostic factor for breast cancer related death in women with node-negative breast cancer. This finding suggests a potential role of ghrelin in breast cancer—both as a prognostic marker but possibly also as a potential therapeutic target. To validate the function of ghrelin in breast cancer, larger population studies are necessary, which also should include functional and genetic studies. With growing understanding in the functionality of ghrelin and the molecular pathways involved, it is a promising therapeutic target.

## References

[1] Kojima M, Hosoda H, Date Y, Nakazato M, Matsuo H, Kangawa K. Ghrelin is a growth-hormone-releasing acylated peptide from stomach. Nature. 1999;402(6762):656–60. Epub 1999/12/22. 10.1038/45230 10604470

[2] Asakawa A, Inui A, Kaga T, Yuzuriha H, Nagata T, Ueno N, et al Ghrelin is an appetite-stimulatory signal from stomach with structural resemblance to motilin. Gastroenterology. 2001;120(2):337–45. Epub 2001/02/13. 11159873 10.1053/gast.2001.22158

[3] Inui A. Ghrelin: an orexigenic and somatotrophic signal from the stomach. Nat Rev Neurosci. 2001;2(8):551–60. Epub 2001/08/03. 10.1038/35086018 11483998

[4] Tack J, Depoortere I, Bisschops R, Delporte C, Coulie B, Meulemans A, et al Influence of ghrelin on interdigestive gastrointestinal motility in humans. Gut. 2006;55(3):327–33. Epub 2005/10/12. 10.1136/gut.2004.060426 16216827 PMC1856079

[5] Korbonits M, Goldstone AP, Gueorguiev M, Grossman AB. Ghrelin—a hormone with multiple functions. Front Neuroendocrinol. 2004;25(1):27–68. Epub 2004/06/09. 10.1016/j.yfrne.2004.03.002 15183037

[6] Cassoni P, Papotti M, Ghe C, Catapano F, Sapino A, Graziani A, et al Identification, characterization, and biological activity of specific receptors for natural (ghrelin) and synthetic growth hormone secretagogues and analogs in human breast carcinomas and cell lines. J Clin Endocrinol Metab. 2001;86(4):1738–45. Epub 2001/04/12. 10.1210/jcem.86.4.7402 11297611

[7] Kahan Z, Arencibia JM, Csernus VJ, Groot K, Kineman RD, Robinson WR, et al Expression of growth hormone-releasing hormone (GHRH) messenger ribonucleic acid and the presence of biologically active GHRH in human breast, endometrial, and ovarian cancers. J Clin Endocrinol Metab. 1999;84(2):582–9. Epub 1999/02/18. 10.1210/jcem.84.2.5487 10022420

[8] Schally AV, Varga JL. Antagonistic Analogs of Growth Hormone-releasing Hormone: New Potential Antitumor Agents. Trends Endocrinol Metab. 1999;10(10):383–91. Epub 1999/11/05. 10542394 10.1016/s1043-2760(99)00209-x

[9] Nakahara K, Hayashida T, Nakazato M, Kojima M, Hosoda H, Kangawa K, et al Effect of chronic treatments with ghrelin on milk secretion in lactating rats. Biochem Biophys Res Commun. 2003;303(3):751–5. Epub 2003/04/03. 12670474 10.1016/s0006-291x(03)00414-5

[10] Yin X, Li Y, Xu G, An W, Zhang W. Ghrelin fluctuation, what determines its production? Acta Biochim Biophys Sin (Shanghai). 2009;41(3):188–97. Epub 2009/03/13. 19280057 10.1093/abbs/gmp001

[11] Chopin L, Walpole C, Seim I, Cunningham P, Murray R, Whiteside E, et al Ghrelin and cancer. Mol Cell Endocrinol. 2011;340(1):65–9. Epub 2011/05/28. 10.1016/j.mce.2011.04.013 21616120

[12] Perks CM, Holly JM. Hormonal mechanisms underlying the relationship between obesity and breast cancer. Endocrinol Metab Clin North Am. 2011;40(3):485–507, vii Epub 2011/09/06. 10.1016/j.ecl.2011.05.010 21889716

[13] Mantzoros C, Petridou E, Dessypris N, Chavelas C, Dalamaga M, Alexe DM, et al Adiponectin and breast cancer risk. J Clin Endocrinol Metab. 2004;89(3):1102–7. Epub 2004/03/06. 10.1210/jc.2003-031804 15001594

[14] Zhang JV, Ren PG, Avsian-Kretchmer O, Luo CW, Rauch R, Klein C, et al Obestatin, a peptide encoded by the ghrelin gene, opposes ghrelin's effects on food intake. Science. 2005;310(5750):996–9. Epub 2005/11/15. 10.1126/science.1117255 16284174

[15] Gourcerol G, Million M, Adelson DW, Wang Y, Wang L, Rivier J, et al Lack of interaction between peripheral injection of CCK and obestatin in the regulation of gastric satiety signaling in rodents. Peptides. 2006;27(11):2811–9. Epub 2006/08/29. 10.1016/j.peptides.2006.07.012 16934368

[16] Chopin LK, Seim I, Walpole CM, Herington AC. The ghrelin axis—does it have an appetite for cancer progression? Endocr Rev. 2012;33(6):849–91. Epub 2012/07/25. 10.1210/er.2011-1007 22826465

[17] Gronberg M, Amini RM, Stridsberg M, Janson ET, Saras J. Neuroendocrine markers are expressed in human mammary glands. Regul Pept. 2010;160(1–3):68–74. Epub 2009/12/29. 10.1016/j.regpep.2009.12.011 20036287

[18] Jeffery PL, Murray RE, Yeh AH, McNamara JF, Duncan RP, Francis GD, et al Expression and function of the ghrelin axis, including a novel preproghrelin isoform, in human breast cancer tissues and cell lines. Endocr Relat Cancer. 2005;12(4):839–50. Epub 2005/12/03. 10.1677/erc.1.00984 16322325

[19] Gronberg M, Fjallskog ML, Jirstrom K, Janson ET. Expression of ghrelin is correlated to a favorable outcome in invasive breast cancer. Acta Oncol. 2012;51(3):386–93. Epub 2011/11/10. 10.3109/0284186X.2011.631576 22067021

[20] Dossus L, McKay JD, Canzian F, Wilkening S, Rinaldi S, Biessy C, et al Polymorphisms of genes coding for ghrelin and its receptor in relation to anthropometry, circulating levels of IGF-I and IGFBP-3, and breast cancer risk: a case-control study nested within the European Prospective Investigation into Cancer and Nutrition (EPIC). Carcinogenesis. 2008;29(7):1360–6. Epub 2008/04/01. 10.1093/carcin/bgn083 18375957

[21] Gahete MD, Rubio A, Cordoba-Chacon J, Gracia-Navarro F, Kineman RD, Avila J, et al Expression of the ghrelin and neurotensin systems is altered in the temporal lobe of Alzheimer's disease patients. J Alzheimers Dis. 2010;22(3):819–28. Epub 2010/09/23. 10.3233/JAD-2010-100873 20858966

[22] Gahete MD, Cordoba-Chacon J, Hergueta-Redondo M, Martinez-Fuentes AJ, Kineman RD, Moreno-Bueno G, et al A novel human ghrelin variant (In1-ghrelin) and ghrelin-O-acyltransferase are overexpressed in breast cancer: potential pathophysiological relevance. PLoS One. 2011;6(8):e23302 Epub 2011/08/11. 10.1371/journal.pone.0023302 21829727 PMC3150424

[23] Garcia JM, Swerdloff R, Wang C, Kyle M, Kipnes M, Biller BM, et al Macimorelin (AEZS-130)-stimulated growth hormone (GH) test: validation of a novel oral stimulation test for the diagnosis of adult GH deficiency. J Clin Endocrinol Metab. 2013;98(6):2422–9. Epub 2013/04/06. 10.1210/jc.2013-1157 23559086 PMC4207947

[24] Costantini VJ, Vicentini E, Sabbatini FM, Valerio E, Lepore S, Tessari M, et al GSK1614343, a novel ghrelin receptor antagonist, produces an unexpected increase of food intake and body weight in rodents and dogs. Neuroendocrinology. 2011;94(2):158–68. Epub 2011/07/23. 10.1159/000328968 21778696

[25] Elston CW, Ellis IO. Pathological prognostic factors in breast cancer. I. The value of histological grade in breast cancer: experience from a large study with long-term follow-up. Histopathology. 1991;19(5):403–10. Epub 1991/11/01. 1757079 10.1111/j.1365-2559.1991.tb00229.x

[26] Gronberg M, Tsolakis AV, Magnusson L, Janson ET, Saras J. Distribution of obestatin and ghrelin in human tissues: immunoreactive cells in the gastrointestinal tract, pancreas, and mammary glands. J Histochem Cytochem. 2008;56(9):793–801. Epub 2008/05/14. 10.1369/jhc.2008.951145 18474938 PMC2516956

[27] Nimeus-Malmstrom E, Koliadi A, Ahlin C, Holmqvist M, Holmberg L, Amini RM, et al Cyclin B1 is a prognostic proliferation marker with a high reproducibility in a population-based lymph node negative breast cancer cohort. Int J Cancer. 2010;127(4):961–7. Epub 2009/12/04. 10.1002/ijc.25091 19957331

[28] Ahlin C, Zhou W, Holmqvist M, Holmberg L, Nilsson C, Jirstrom K, et al Cyclin A is a proliferative marker with good prognostic value in node-negative breast cancer. Cancer Epidemiol Biomarkers Prev. 2009;18(9):2501–6. Epub 2009/08/27. 10.1158/1055-9965.EPI-09-0169 19706846

[29] VanderWeele TJ, Hernan MA, Robins JM. Causal directed acyclic graphs and the direction of unmeasured confounding bias. Epidemiology. 2008;19(5):720–8. Epub 2008/07/18. 10.1097/EDE.0b013e3181810e29 18633331 PMC4242711

[30] Pearl J. Causality: models, reasoning and inference. Cambridge: Cambridge University Press; 2000.

[31] Spirtes P G C, Scheines R Causation, Prediction, and Search. Cambridge: MIT Press; 2000.

[32] Altman D. Practical Statistics för Medical Research. London, UK: Chapman & Hall; 1991.

[33] Ahlin C, Aaltonen K, Amini RM, Nevanlinna H, Fjallskog ML, Blomqvist C. Ki67 and cyclin A as prognostic factors in early breast cancer. What are the optimal cut-off values? Histopathology. 2007;51(4):491–8. Epub 2007/08/23. 10.1111/j.1365-2559.2007.02798.x 17711446

[34] Seim I, Collet C, Herington AC, Chopin LK. Revised genomic structure of the human ghrelin gene and identification of novel exons, alternative splice variants and natural antisense transcripts. BMC Genomics. 2007;8:298 Epub 2007/08/31. 10.1186/1471-2164-8-298 17727735 PMC2014779

[35] Nass R, Gaylinn BD, Thorner MO. The ghrelin axis in disease: potential therapeutic indications. Mol Cell Endocrinol. 2011;340(1):106–10. Epub 2011/03/02. 10.1016/j.mce.2011.02.010 21356273 PMC3114265

[36] Seim I, Herington AC, Chopin LK. New insights into the molecular complexity of the ghrelin gene locus. Cytokine Growth Factor Rev. 2009;20(4):297–304. Epub 2009/08/12. 10.1016/j.cytogfr.2009.07.006 19665916

